# Dental status, salivary flow, and sociodemographic aspects in Sheehan Syndrome patients

**DOI:** 10.4317/medoral.22377

**Published:** 2018-06-21

**Authors:** Davi-de-Sá Cavalcante, Ana-Rosa Pinto-Quidute, Manoel-Ricardo Alves-Martins, Andrea-Silva Walter-de-Aguiar, Adília-Mirela-Pereira Lima-Cid, Paulo-Goberlânio-de Barros Silva, Rocharles-Fontenele Cavalcante, Fábio-Wildson-Gurgel Costa

**Affiliations:** 1 DDS, MSc candidate, Division of Oral Radiology, Postgraduate Program in Dentistry, Federal University of Ceará, Fortaleza, Brazil; 2 DDS, MSc, PhD, Division of Endocrinology and Diabetes, Walter Cantídio University Hospital, Fortaleza, Brazil; 3 DDS, MSc, PhD, Division of Clinical Dentistry, Postgraduate Program in Dentistry, Federal University of Ceará, Fortaleza, Brazil; 4 Graduate student, Division of Oral Radiology, Postgraduate Program in Dentistry, Federal University of Ceará, Fortaleza, Brazil; 5 DDS, MSc, PhD, Division of Oral Pathology, Postgraduate Program in Dentistry, Federal University of Ceará, Fortaleza, Brazil; 6 DDS, MSc candidate, Division of Oral Radiology, Piracicaba Dental School, University of Campinas, São Paulo, Brazil

## Abstract

**Background:**

Sheehan’s syndrome (SS) is one of the leading causes of hypopituitarism in developing countries. It occurs after postpartum necrosis of the pituitary gland, and it is considered a significant public health problem. This paper, apparently unpublished, aimed to perform an analysis on oral aspects in patients with SS.

**Material and Methods:**

A cross-sectional study was performed with 23 women diagnosed with SS at the Division of Endocrinology and Diabetes (Walter Cantídio University Hospital, Fortaleza, Brazil).

**Results:**

Data on sociodemographic, dental and salivary flow aspects were collected through a clinical approach and a panoramic radiograph request. The mean age was 64 ± 11.5 years old, with the sample consisting mainly of married women (56.5%), socioeconomic class C2 or D / E (78.2%) and years of education up to 8 years (69.5%). The presence of horizontal bone loss (*p*<0.001) and bilateral pneumatization of the maxillary sinus (*p*=0.015) were significant data. The mean number of absent teeth considering all subjects was 23.17±9.7, being statistically significant (*p*<0.001). In relation to age, the mean number of missing teeth was higher in individuals over 65 years old (*p*=0.048). Reduced salivary flow was observed in 78.3% of the patients. In a bivariate analysis, considering the outcome variables “missing teeth” and “reduced salivary flow”, it was observed that economic class (*p*< 0.001), family income (0.037) and maxillary sinus pneumatization (0.032) were statistically significant.

**Conclusions:**

In brief, patients with SS showed severe teeth loss, reduced salivary flow, and low educational status. This study addressed important aspects regarding oral findings in SS and highlighted the importance of researches in oral medicine.

** Key words:**Sheehan’s Syndrome, hypopituitarism, tooth loss, salivary flow.

## Introduction

The stomatognathic system has been considered a field for the manifestation of endocrine system disorders (1). Sheehan syndrome (SS), also known as postpartum pituitary necrosis (2), is an uncommon condition characterized by partial or total necrosis of the pituitary gland. Some clinical findings include agalactia, amenorrhea, asthenia, hypoglycemia, hypotension, hydroelectrolytic disorders, gain of weight, and constipation. The presence of multiple hormonal deficiencies leads to impairment of bone microarchitecture, which can cause osteopenia and even osteoporosis (3,4).

Although there are no studies on the oral aspects in SS, the local effect of pituitary hormones deficiency on bone and dental tissues has been described. Reduction of estrogen levels may affect oral cavity epithelium, salivary gland function, and propensity for inflammatory processes related to periodontal tissues (5). The onset of inflammation in the oral cavity has a higher probability of further tooth loss (6). Since tooth pathologies have been associated with a more significant decline in functional capacity, physical imbalance, altered cognitive function, and impairment in the performance of communicative social skills (7), it reinforces the importance of studies evaluating oral aspects in patients with chronic hypopituitarism. In this context, this study aimed to evaluate tooth loss, sociodemographic data, and salivary flow in Brazilian SS patients. To date, there are no published studies focusing on the oral health of SS.

## Material and Methods

A cross-sectional study was performed with patients diagnosed with SS from the Endocrinology and Diabetes Division at the Walter Cantídio University Hospital (Fortaleza, Ceará, Brazil), who had been in clinical follow-up for more than ten years. The research protocol was evaluated by the Human Research Ethics Committee of the Federal University of Ceará (approval number # 983,022).

SS is considered a rare disease, with an incidence of 0.2 to 2.8 cases per 100,000 women in developed countries (8). Thus, the present sample was of convenience. Patients with a previously confirmed diagnosis of SS, under periodic medical follow-up, and those that agreed to participate in this study after reading and signing an informed consent form were included. Patients who met the eligibility criteria were submitted to an anamnesis, imaging exam, and salivary flow assessment. Sociodemographic aspects, dental functional status, salivary flow and medical data (age and time of diagnosis, the age of last childbirth, presence/absence of agalactia, amenorrhea, obstetric history of postpartum hemorrhage, and hormonal deficiencies) were evaluated.

Age was grouped into two categories (up to 65 and over 65 years). Marital status was categorized as follows: married, single, divorced, and widowed. Socioeconomic profile followed the Brazilian Economic Classification Criteria (http://www.abep.org/criterio-brasil), and the number of obtained points stratified the individual into classes, from A1 (the most favored) to E (the least favored). Socioeconomic status was categorized according to Noce *et al.* (9), which considered the years of education and the monthly family income.

The dental functional status was obtained through the evaluation of the number of teeth in digital panoramic radiographs, which were classified according to Sato *et al.* (9) as edentulism and teeth groups (≥ 20, 10-19, 1-9). It was used the Kodak K9000 3D equipment (Kodak Dental Systems, Carestream Health, Rochester, NY, USA). Additionally, presence/absence of horizontal bone loss, maxillary sinus pneumatization (sinus wall depression toward maxillary alveolar ridge, rendering an imaging aspect of increased sinus space), degenerative condylar alterations, and styloid process elongation were recorded. Before obtaining the panoramic radiograph, a saliva sample was collected for each participant between 8:00 and 11:00 am during a 5-minute period (salivary flow as mL/min).

The Statistical Package for the Social Sciences software (version 20.0 for Windows®) was used. Descriptive statistics and data frequency were recorded. Also, Chi-square or Fisher’s exact and Mann-Whitney tests were used (significance level of 5%).

## Results

A sample of 23 patients diagnosed with SS was evaluated in this study, showing a mean age of 64±11.5 years (47-79 years). Concerning the medical data, the mean age at the time the diagnosis was 40.38±10.53 years, while the mean time of disease diagnosis delay was 10.92±8.22 years. The mean age at the time of the last childbirth was 29.53±6.61 years. Also, it was recorded agalactia (46.15%), amenorrhea (53.84%), and adynamia (61.54%). During a 10-year clinical follow-up, 92.31% were diagnosed with hypothyroidism, and 100% had glucocorticoid replacement. Therefore, all patients had more than 1 hormone deficiency. No patient had a replacement of the somatotropic axis, due to the following factors: socioeconomic status, medication unavailability at the time of diagnosis in the public service, and no indication of replacement for patients ≥ 60 years or older according to the Brazilian Ministry of Health protocol.

According to  Table 1, 51.8% of the patients were up to 65 years at the time of dental evaluation, and the majority of the sample consisted of married subjects (56.5%). Low socioeconomic status was observed among SS patients, since 78,2% belonged to the category C2 or D/E, and about 70% of the patients had up to 8 years of schooling and low family income. The horizontal alveolar bone loss was observed in all patients (*p*<0.001), the most prevalent pneumatization of the maxillary sinus was bilateral (*p*=0.015), and the absence of degenerative changes in the mandibular condyle and normal styloid process were commonly observed (*p*=0.017 and *p*=0.003, respectively).

**Table 1:**
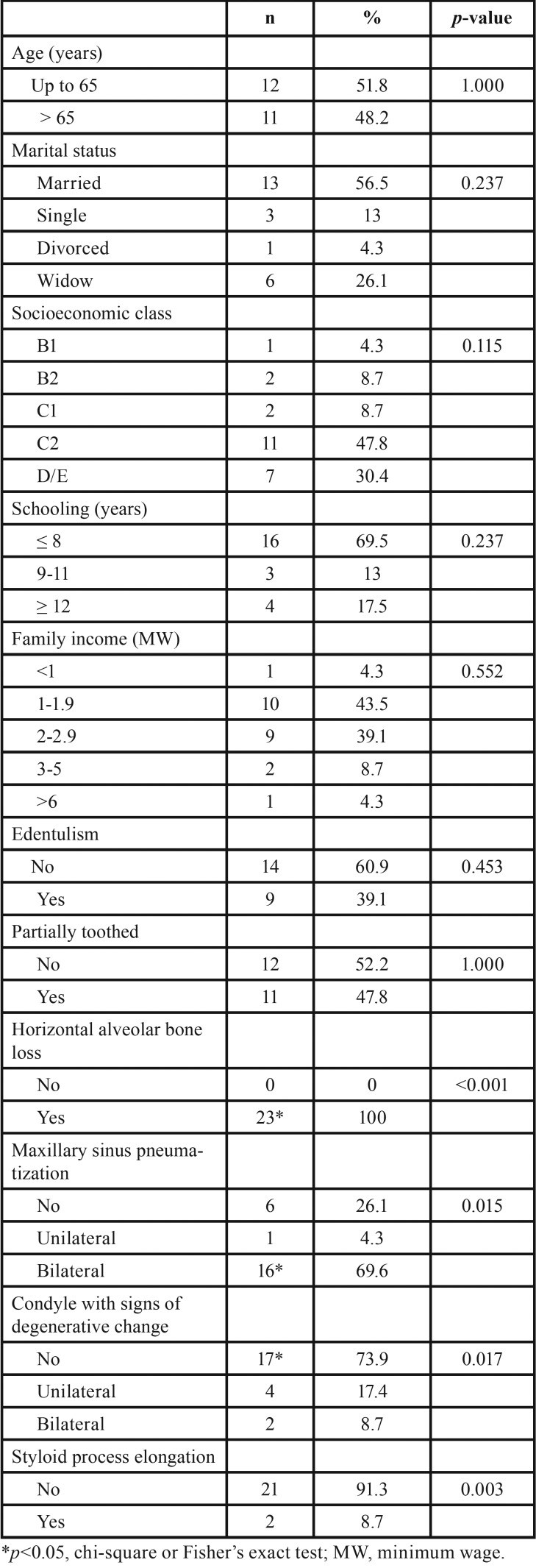
Sample characterization regarding sociodemographic data and imaging variables.

**Figure 1:**
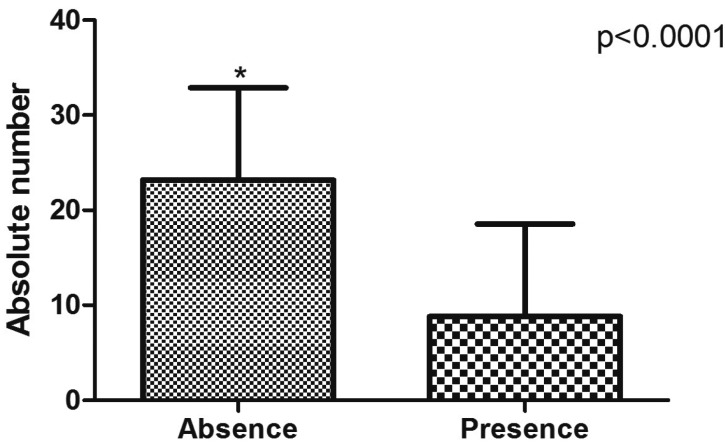
Mean number of missing teeth in SS sample. Asterisk (*) indicates statistical significance (Mann-Whitney test).

Regarding tooth status, 47% of the patients were partially dentate ( Table 1). The mean number of absent teeth was 23.17 ± 9.7, which was statistically significant (*p*<0.0001; Fig. 1). According to  Table 2, the maxilla concentrated 53.45% of teeth loss. In jaws, missing teeth were mainly in the posterior mandible (34.3%), followed by posterior maxilla (33.53%), anterior maxilla (19.92%), and anterior mandible (12.26%). The mean number of missing teeth in patients with maxillary sinus pneumatization ( Table 2) was statistically significantly (*p*=0.001). The prevalence of maxillary sinus pneumatization was statistically significant (*p*=0.006) in patients with 10-19 (11.7; odds ratio of 0.3-422.5) and ≥20 (43.4; odds ratio of 1.7-1121.0) missing teeth.

**Table 2:**
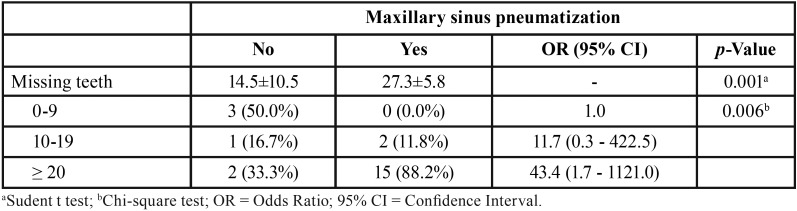
Teeth loss in patients with maxillary sinus pneumatization.

**Figure 2:**
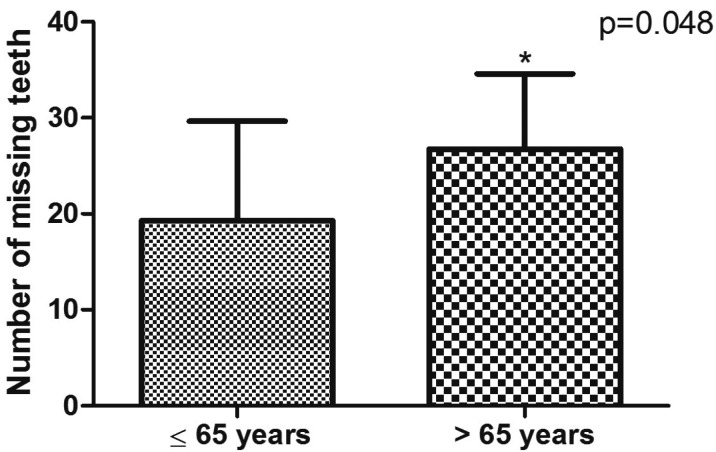
Mean number of missing teeth considering individuals ≤65 years versus >65 years. Asterisk (*) indicates statistical significance (Mann-Whitney test).

Figure 2 shows that the mean number of missing teeth was statistically higher (*p*=0.048) in individuals over 65 years. In subjects up to 65 years ( Table 3), tooth loss was more prevalent in the posterior mandible (17.82%) followed by posterior maxilla (15.71%). The patients over 65 years showed a higher prevalence of tooth loss in the posterior maxilla (17.82%), followed by posterior mandible (16.48%).

**Table 3:**
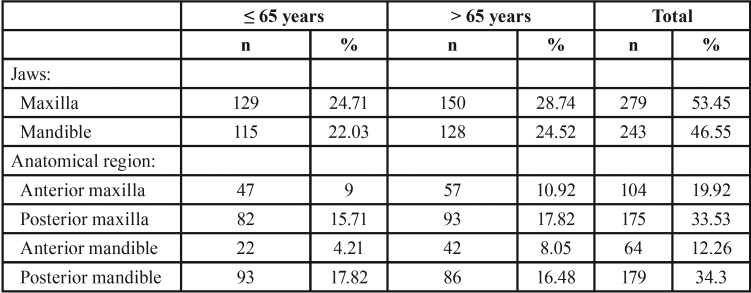
Anatomical distribution of missing teeth (n=522) according to age.

The mean salivary flow value was 0.14±0.16 in individuals over 65 years of age and 0.2±0.31 in individuals aged up to 65 years. In 78.3% of the sample, reduced salivary flow was found. Regarding reduced salivary flow, 72.22% (n=13) presented very low values and 27.78% (n=5) presented low values. Also, in a bivariate analysis considering the outcome variables “missing teeth” and “reduced salivary flow” ( Table 4), it was observed that economic status (*p*<0.001), family income (*p*=0.037), and maxillary sinus pneumatization (*p*=0.032) were statistically significant variables.

**Table 4:**
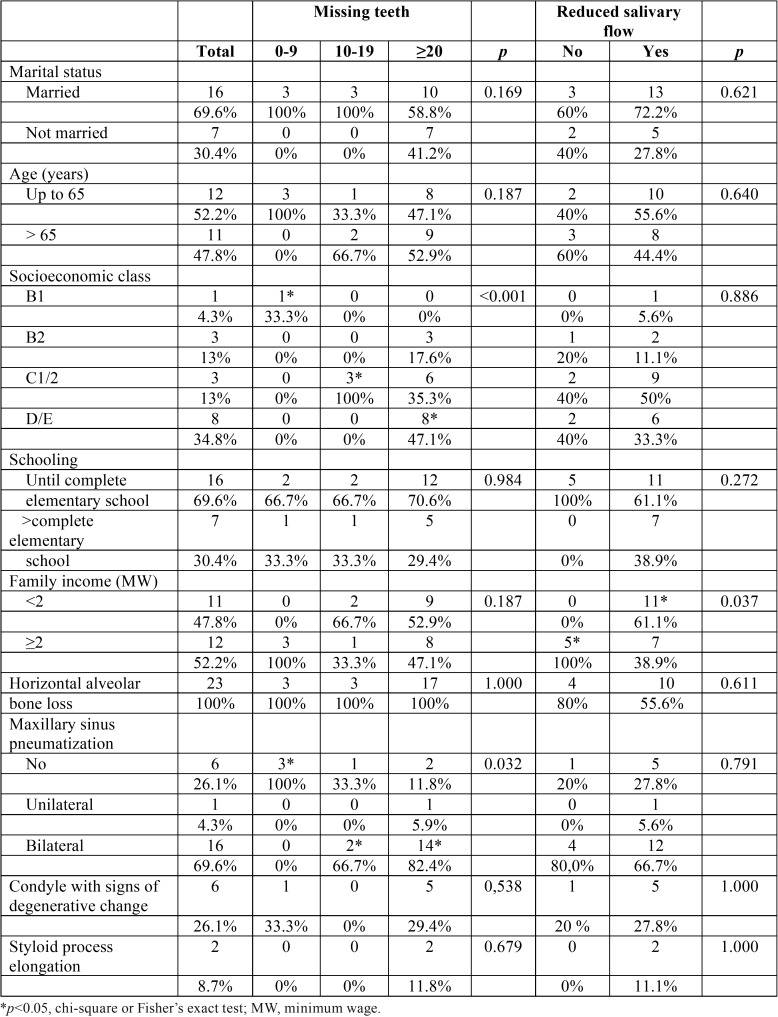
Influence of the outcome variables “missing teeth” and “reduced salivary flow” on study variables.

## Discussion

The study of systemic diseases, especially hypopituitarism, can be considered a valuable field of research in oral medicine. In this context, SS should be investigated because it is an endocrinopathy still observed in developing countries such as Brazil, but there are no published studies on its oral aspects to date.

The present study investigated the relationship between tooth loss, sociodemographic variables, and salivary flow in a sample of SS subjects, which were more prevalent in the sixth decade of life. This data agrees with Diri *et al.* (10), which reported patients with 63.2±12.5 years, as well as with the study of Dökmetaş *et al.* (11) that reported an average age of 60.1 years in a sample of 20 patients. However, a study of 28 patients diagnosed with SS in the period between 1982 and 2002 in Turkey showed individuals with a mean age of 48.2±10.5 years (12).

The presence teeth is considered a reliable indicator of oral and systemic health (13), and some authors have observed that longevity/life expectancy may be affected by the number of teeth present and that tooth loss is a predictive factor shortening of longevity (14). A dentition with various dental absences attenuates masticatory efficiency and causes a subsequent high limiting food selection with a low diet in fruits, vegetables and other essential nutrients (15). Such aspects reinforce the importance of the present study when analyzing the dental status in a group of patients with endocrinopathy still present in developing countries. It was observed that the individuals affected by SS had a high number of dental losses, with the majority having 20 or more missing teeth. In this context, in a study investigating the relationship between the number and position of teeth with satisfaction with the oral cavity, it was found that at least 20 natural teeth are required to obtain satisfactory aesthetic function and function (16). Hirotomi *et al.* (17) observed in a 5-year longitudinal study that individuals with 20 or more teeth had a lower mortality rate (2.5%) than individuals with up to 19 teeth (6.1%), which was statistically significant. Hayasaka *et al.* (18) showed an inverse dose-response relationship between the number of remaining teeth and mortality, and individuals with 10 to 19 teeth presented a relative risk of 1.16 compared to individuals with 20 or more teeth.

Over the last few years, sociodemographic variables have been considered important factors are contributing to tooth loss (19), with emphasis on educational and socioeconomic levels (14). According to Rozier *et al.* (20), low socioeconomic status was the most consistent predictive factor of missing teeth in the US population.

In Brazil, a longitudinal study that used the Gini Index to measure socioeconomic inequality showed that the increase in this index was associated with a higher prevalence of severe missing teeth and loss of a functional dentition, which is considered when there were at least 20 natural teeth (21). Presently, patients with SS showed low socioeconomic status. This finding was similar to Silva *et al.* (22) study that used the Economic Classification Criteria Brazil. These authors observed a significant association between socioeconomic status and the number remaining natural teeth, evidencing that individuals with high socioeconomic status had a higher number of permanent teeth when compared to lower socioeconomic status. Also, the present data corroborate the socioeconomic profile of patients with SS described in the literature. Famuyiwa *et al.* (23) identified 11 patients with SS over a 5-year period from a university hospital in Nigeria and observed that about 64% of the sample belonged to a low socioeconomic class.

The impairment of salivary function is associated with teeth loss due to a high risk of caries and periodontal disease (24). In the present study, it was observed that patients with SS had low salivary flow in the majority, representing about 80% of the analyzed sample. In this specific group of patients, the majority had significant low values of salivary flow. These findings corroborate the high percentage of missing teeth, which was a statistically significant result. Marques *et al.* (25) investigated predictive factors of tooth loss among Brazilian adults and found that the low salivary flow represented an independent outcome variable. In a logistic regression model controlling marital status, race, and socioeconomic status performed by Caplan, Hunt (24) among 818 patients aged at least 65 years, a statistically significant association was observed between low salivary flow and dental loss.

Gokalp *et al.* (4) highlighted the importance of hypogonadotropic hypogonadism in the development of osteoporosis in SS patients. These authors conclude that growth hormone has a significant effect on bone metabolism and plays a crucial role in maintaining bone mass in adults by regulating bone remodeling. In this context, such systemic findings reinforce the data found in the present study, especially the severe dental loss observed, which also reflected a significant prevalence of horizontal bone loss and maxillary sinus pneumatization. It is noteworthy that in our research there was no adequate replacement of the gonadal axis nor of the somatotropic axis by previously reported factors, being patients that due to delayed diagnosis was submitted to many years of hypoestrogenism. Chihaoui *et al.* (26) in a study of 60 patients diagnosed with SS concluded that reduced bone mineral density was a finding frequently observed in such individuals. Such result inserted in the context of the present study is important because of the existence of an association between low bone mineral density and tooth loss and maxillomandibular alveolar bone loss (27).

## Conclusions

In summary, the present study evidenced oral cavity-related fidings reflecting the chronic hypopituitarism related to SS. It was observed a sample represented by adult women, mainly in the sixth decade of life, who presented low educational and socioeconomic levels, reduced salivary flow, and severe teeth missing, which could be associated with a decline in functional capacity of women affected by SS.
